# 5-azacytidine induces transcriptome changes in *Escherichia coli* via DNA methylation-dependent and DNA methylation-independent mechanisms

**DOI:** 10.1186/s12866-016-0741-4

**Published:** 2016-06-27

**Authors:** Kevin T. Militello, Robert D. Simon, Alexandra H. Mandarano, Anthony DiNatale, Stacy M. Hennick, Justine C. Lazatin, Sarah Cantatore

**Affiliations:** State University of New York at Geneseo, ISC 357, 1 College Circle, Geneseo, NY 14454 USA; Cornell University, Ithaca, NY 14853 USA

**Keywords:** Stationary phase, 5-azacytidine, DNA methylation inhibitor, DNA methylation, 5-methylcytosine, Dcm, RpoS, *Escherichia coli*, Sodium bisulfite sequencing, DNA microarray

## Abstract

**Background:**

*Escherichia coli* K-12 strains contain DNA cytosine methyltransferase (Dcm), which generates 5-methylcytosine at 5′CCWGG3′ sites. Although the role of 5-methylcytosine in eukaryotic gene expression is relatively well described, the role of 5-methylcytosine in bacterial gene expression is largely unknown.

**Results:**

To identify genes that are controlled by 5-methylcytosine in *E. coli*, we compared the transcriptomes of cells grown in the absence and presence of the DNA methylation inhibitor 5-azacytidine. We observed expression changes for 63 genes. The majority of the gene expression changes occurred at early stationary phase and were up-regulations. To identify gene expression changes due to a loss of DNA methylation, we compared the expression of selected genes in a wild-type and *dcm* knockout strain via reverse transcription quantitative PCR.

**Conclusions:**

Our data indicate that 5-azacytidine can influence gene expression by at least two distinct mechanisms: DNA methylation loss and a mechanism that is independent of DNA methylation loss. In addition, we have identified new targets of 5-methylcytosine-mediated regulation of gene expression. In summary, our data indicate that 5-azacytidine impacts the composition of the bacterial transcriptome, and the primary effect is increased gene expression at early stationary phase.

**Electronic supplementary material:**

The online version of this article (doi:10.1186/s12866-016-0741-4) contains supplementary material, which is available to authorized users.

## Background

The modified DNA base 5-methylcytosine (5-MeC) plays an important role in transcriptional regulation in higher eukaryotes. The presence of 5-MeC in eukaryotic promoters and CpG islands is generally repressive for transcription, whereas 5-MeC in gene bodies is positively correlated with transcription [1]. Bacteria, such as *E. coli*, contain 5-MeC [2]. In *E. coli* K-12 strains, the only known cytosine-5 DNA methyltransferase is DNA cytosine methyltransferase (Dcm) [3, 4]. Dcm methylates the second cytosine in 5′CCWGG3′ sequences [3]. The *dcm* gene is in an operon with the *vsr* gene which codes for a protein that repairs T:G mismatches caused by deamination of 5-MeC [5–7]. The original function elucidated for Dcm was in restriction enzyme biology where Dcm promotes the loss of plasmids containing the EcoRII restriction enzyme gene (which cleaves 5′CCWGG3′ sites) and protects cells from post-segregational killing by the EcoRII restriction enzyme [8, 9]. In addition, Dcm protects phage lambda against DNA cleavage when EcoRII is introduced into the cell [10]. However, Dcm is a solitary methyltransferase without a cognate restriction enzyme in K-12 cells. Other roles for Dcm are certainly possible.

Based on the important role of 5-MeC in eukaryotic transcription and the fact that there is little known about the relationship between 5-MeC and gene expression in bacteria, Dcm has been recently evaluated for an impact on the composition of the *E. coli* transcriptome. Our group has demonstrated that two ribosomal protein genes and the drug resistance transporter gene *sugE* are upregulated in the absence of the *dcm* gene at early stationary phase via reverse-transcription quantitative PCR (RT-qPCR) [11, 12]. Kahramanoglou *et al*. demonstrated that there are gene expression changes in *dcm* knockout cells using DNA microarrays, and most changes are at stationary phase [13]. Taken together, these data suggest that Dcm influences the transcriptome. As the only known function of Dcm is cytosine DNA methylation, the simplest model is that Dcm mediates gene expression changes via the generation of 5-MeC. It is noteworthy that some DNA methyltransferases can methylate tRNA and influence gene expression via a DNA-methylation independent mechanism [14–16].

In order to test the model that Dcm-mediated cytosine DNA methylation directly influences gene expression in *E. coli* and identify new genes impacted by DNA methylation, we analyzed the *E. coli* transcriptome in the absence and presence of 5-azacytidine (5-azaC) treatment. 5-azaC is a nucleoside analog that is used clinically to treat myelodysplastic syndromes [17]. 5-azaC is phosphorylated upon cell entry and incorporated into both RNA and DNA [18, 19]. When 5-azaC is incorporated into DNA, cytosine-5 DNA methyltransferases become covalently trapped on the DNA and are degraded, and this limits the amount of enzyme available for the generation of 5-MeC [18, 19]. Thus, 5-azaC is a cytosine DNA methylation inhibitor. It is important to note that 5-azaC has effects on the cell beyond blocking DNA methylation. For example, 5-azaC can induce the SOS response [20, 21], induce DNA mutations [22], block translation [23], and block RNA methylation [24]. Thus, the physiology of 5-azaC treated cells is not identical to cells lacking cytosine DNA methyltransferases. Although 5-azaC has been routinely used to demethylate DNA in a variety of eukaryotes to assess the consequences of cytosine DNA methylation loss [25, 26], this is the first report of the response of the entire transcriptome to 5-azaC in a bacterial organism.

## Results

### Effects of 5-azaC on global DNA methylation levels

**Fig. 1:**
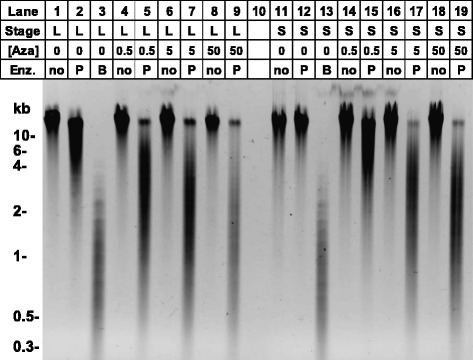
The effect of 5-azaC on global DNA methylation levels in *Escherichia coli*. *E. coli* wild-type strain BW25113 was grown in LB at 37 °C in the absence of 5-azaC, the presence of 0.5 μg/mL 5-azaC, 5 μg/mL 5-azaC, and 50 μg/mL 5-azaC. At two hours (logarithmic phase, L) and eight hours (early stationary phase, S), genomic DNA was isolated. DNA was incubated in the absence of restriction enzyme, presence of BstNI (B) (not blocked by Dcm methylation), or presence of PspGI (P) (blocked by Dcm methylation). DNA samples were analyzed by agarose gel electrophoresis and ethidium bromide staining

First, we determined the concentration dependence of DNA methylation inhibition by 5-azaC using digestion of DNA with the restriction enzyme isoschizomers BstNI and PspGI (Fig. 1). Both enzymes cut DNA at Dcm recognition sites (5′CCWGG3′), but PspGI is blocked by Dcm-mediated methylation of the second cytosine. In the absence of 5-azaC, DNA from early stationary phase cells was largely resistant to PspGI indicating that the DNA is heavily methylated at this stage. At early logarithmic stage, DNA was slightly sensitive to PspGI, indicating that most but not all 5′CCWGG3′ sites are methylated. These data are consistent with our current results (Fig. 3) indicating that there is 100 % methylation of a single *osmE* 5′CCWGG3′ site analyzed by sodium bisulfite sequencing from early stationary phase DNA and 93.3 % methylation of a single *osmE* 5′CCWGG3′ site analyzed by sodium bisulfite sequencing from logarithmic phase DNA. The data are also consistent with previously published results indicating stationary phase DNA is heavily methylated, whereas logarithmic phase DNA is methylated but has some unmethylated sites [13, 27]. It is currently unclear if the lack of 100 % methylation at logarithmic phase is due to active DNA replication where newly synthesized DNA is unmethylated or regulation of DNA methylation. When DNA was isolated from cells incubated in the presence of 5-azaC, the DNA was more sensitive to digestion with PspGI, indicating 5-azaC inhibits DNA methylation at all concentrations used at both phases. There was an increase in PspGI sensitivity as the concentration moved from 0.5 μg/mL 5-azaC to 5 μg/mL 5-azaC, especially in early stationary phase DNA.

### Effects of 5-azaC on bacterial growth

**Fig. 2:**
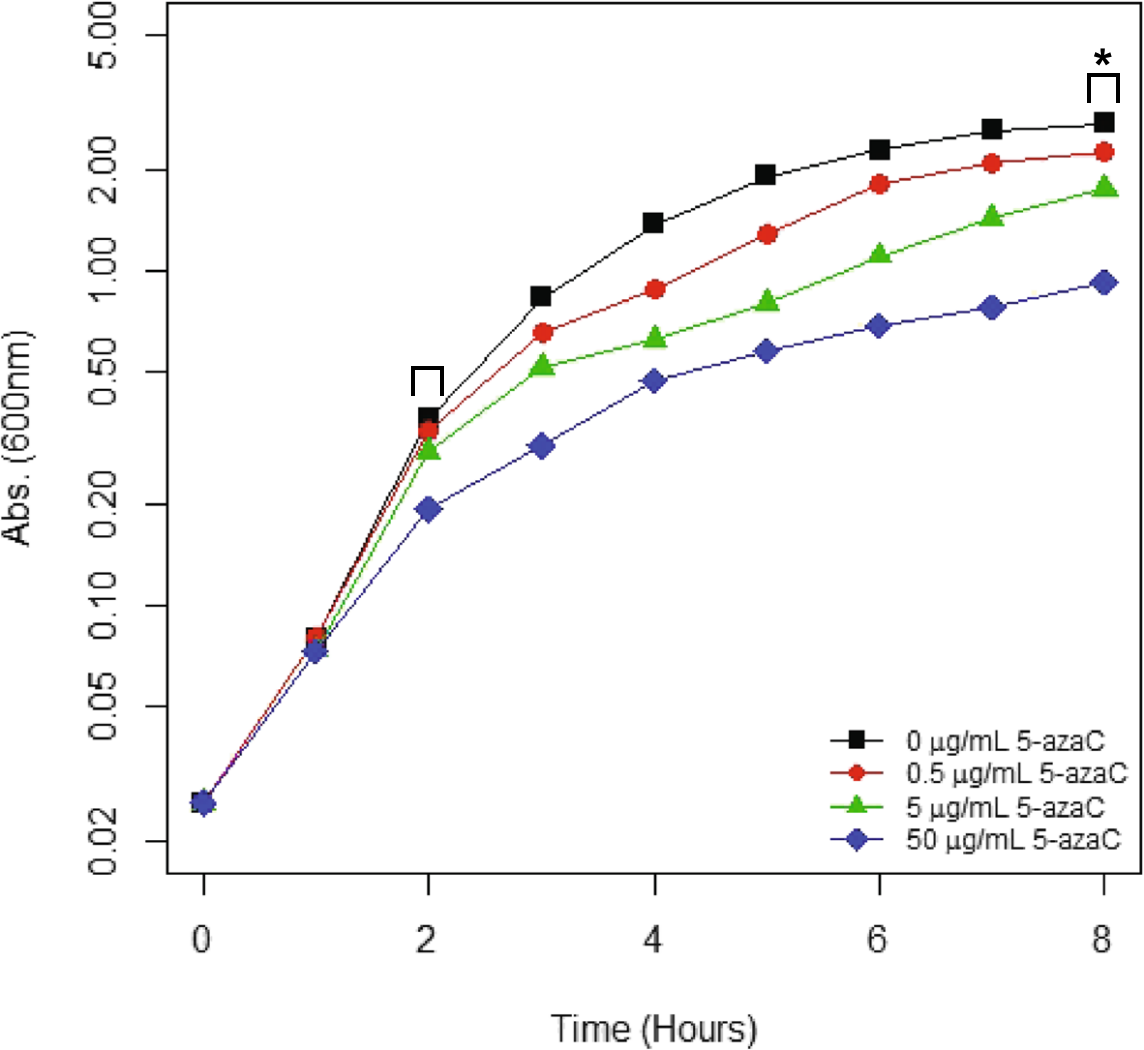
The effect of 5-azaC on *Escherichia coli* growth in liquid media. *E. coli* wild-type strain BW25113 was grown in LB at 37 °C in the absence of 5-azaC (black squares), the presence of 0.5 μg/mL 5-azaC (red circles), 5 μg/mL 5-azaC (green triangles) and 50 μg/mL 5-azaC (blue diamonds) for eight hours. At every hour, each culture was analyzed by spectrophotometry at 600 nm. The data are from three biological replicates (*n* = 3). Error bars representing standard deviation are not shown to maintain clarity of the four lines. Brackets indicate time points of RNA isolation for microarray analysis, and data analyzed by ANOVA. The asterisk indicates a p-value of <0.05

We conducted experiments to determine the effects of 5-azaC on the kinetics of *E. coli* growth (Fig. 2). Bacterial cells were grown in the absence of 5-azaC and three concentrations of the drug as described previously. There was little effect of 5-azaC on bacterial growth for the first few hours. At two hours, which represents logarithmic phase and one time point for the DNA microarray analysis, ANOVA indicated that there is not a statistically significant difference in A_600_ readings between the four cultures (*p* = 8.3E-2). This is likely due to the fact that 5-azaC has to be transported into the cells, phosphorylated, and incorporated into nucleic acids before it is active. After 8 h of growth in 5-azaC (early stationary phase), ANOVA indicated there was a statistically significant difference in A_600_ readings (*p* = 9.9E-5). Post hoc analysis indicated that there was no difference in A_600_ readings between untreated cultures and cultures grown in 0.5 μg/mL 5-azaC (*p* = 1.4E-1). However, there was a statistically significant difference between untreated cultures and cultures grown in 5 μg/mL 5-azaC (*p* = 4.7E-3) and 50 μg/mL 5-azaC (*p* = 7.6E-5). In summary, the data are consistent with previous experiments indicating that high concentrations of 5-azaC can inhibit bacterial growth [28–30]. Based on the PspGI digestion data and the growth curve data above, we chose 5 μg/mL as our working concentration of 5-azaC. At 5 μg/mL, there is an obvious reduction of DNA methylation, and the reduction is greater than that observed using 0.5 μg/mL 5-azaC. With respect to growth defects, the growth defect with 5 μg/mL 5-azaC is less than that observed using 50 μg/mL 5-azaC. A severe growth defect could compromise the interpretation of downstream DNA microarray experiments.

### Effects of 5-azaC on site specific methylation

**Fig. 3:**
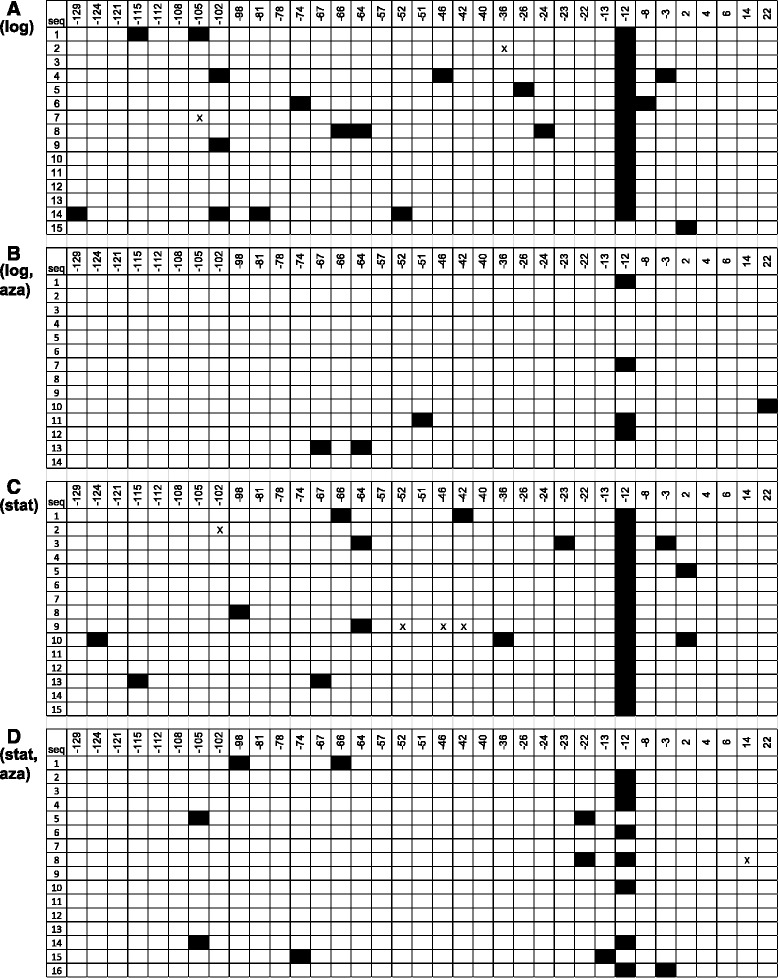
5-azaC reduces cytosine DNA methylation of the *osmE* promoter in *Escherichia coli. E. coli* wild-type strain BW25113 was grown in LB at 37 °C in the absence of 5-azaC and presence of 5 μg/mL 5-azaC. DNA was isolated after two hours (logarithmic phase) and eight hours (early stationary phase). DNA samples were treated with sodium bisulfite. The *osmE* locus was amplified by PCR using sodium bisulfite-treated DNA as a template. PCR products were inserted into the plasmid pGEM-T Easy, and analyzed by Sanger sequencing. **a**) logarithmic phase DNA in untreated cells, **b**) logarithmic phase DNA in 5-azaC treated cells, **c**) early stationary phase DNA in untreated cells, and **d**) early stationary phase DNA in 5-azaC treated cells. The numbers represent the position of the cytosines with respect to the transcription start site (+1). The cytosines in the 5′CCWGG3′ site are at positions −13 and −12. Open boxes represent Cs in the *osmE* sequence (Ts after bisulfite sequencing), and closed boxes represent 5-MeCs in the *osmE* sequence (Cs after bisulfite sequencing). Rows represent different clones. Xs represent ambiguous sequence information

The PspGI assay described above to monitor DNA methylation is primarily qualitative. To better quantify the ability of 5-azaC to block DNA methylation in *Escherichia coli*, we analyzed the *osmE* promoter region for 5-MeC from cells grown in the absence and presence of 5 μg/mL 5-azaC using sodium bisulfite sequencing. Treatment of DNA with sodium bisulfite causes the deamination of cytosines to uracils, but 5-MeCs are not deaminated and remain as cytosines in DNA sequencing reactions [31]. *OsmE* was chosen as it contains one 5′CCWGG3′ site in its promoter. The sodium bisulfite sequencing data are shown in Fig. 3. At the second cytosine in the single 5′CCWGG3′ site in the DNA analyzed, 93.3 % of the logarithmic phase cytosines and 100 % of the early stationary phase cytosines were resistant to bisulfite conversion. At the remaining 34 non-5′CCWGG3′ sites, only 3.3 % of the logarithmic phase cytosines and 2.6 % of the early stationary phase cytosines were resistant to bisulfite conversion. The data are consistent with the model that Dcm methylates 5′CCWGG3′ sites. Low levels of non-5′CCWGG3′ methylation are most easily explained by a very low level of non-conversion of cytosines to uracil, which is always observed in bisulfite sequencing reactions. However, it is not possible to rule out low levels of non-5′CCWGG3′ methylation. In the presence of 5-azaC, the level of methylation at the 5′CCWGG3′ site was reduced from 93.3 % to 28.6 % at logarithmic phase and from 100 % to 50 % at early stationary phase. The potency of 5-azaC at early logarithmic phase is likely due to better incorporation of the drug into DNA during DNA replication at this stage, yet there is inhibition at early stationary phase as well. In summary, the data demonstrate that 5 μg/mL 5-azaC blocks, but does not eliminate, DNA methylation at 5′CCWGG3′ sites in *E. coli*.

### Transcriptome changes in 5-azaC treated cells

We compared *E. coli* transcriptomes in the absence and presence of 5-azaC using DNA microarray analysis of logarithmic phase and early stationary phase RNAs (Additional files 1 and 2). Overall, we detected six up-regulated genes and one down-regulated gene at logarithmic phase (Table 1), and 44 up-regulated genes and 12 down-regulated genes at early stationary phase (Table 2). To confirm the validity of the microarray experiments, we analyzed expression of the *recA* and *rsmI* genes in 5-azaC treated cells via RT-qPCR (Table 3). The RT-qPCR experiments confirmed the direction of the changes observed in the microarray experiments; the RT-qPCR fold-changes were slightly higher.

**Table 1:** Gene expression changes in logarithmic phase, 5-azacytidine-treated *Escherichia coli* cells

Gene ID	Name	Description	fold change (log2)	p
b2616	*recN*	Recombination and repair	2.76	2.52E-04
b3645	*dinD*	DNA-damage-inducible protein, function unknown	2.24	2.20E-05
b3146	*rsmI*	16S rRNA C1402 2′-O-ribose methyltransferase, SAM-dependent	1.82	5.84E-05
b0231	*dinB*	DNA polymerase IV, capable of translesion synthesis; overexpression enhances mutagenesis; mediates targeted mutagenesis by 4-NQO; intrinsic AP lyase activity	1.52	2.28E-04
b3832	*rmuC*	DNA recombination protein; mutants have elevated recombination at microhomologies	1.30	3.36E-05
b0799	*dinG*	ATP-dependent DNA helicase; putative repair and recombination enzyme, monomeric	1.05	1.74E-04
b2876	*yqeC*	Putative selenium-dependent hydroxylase accessory protein	−1.13	7.92E-05

**Table 2:** Gene expression changes in early stationary phase, 5-azacytidine-treated *Escherichia coli* cells

Gene ID	Name	Description	fold-change (log2)	p
b1557	*cspB*	Cold shock protein, Qin prophage; cold shock inducible	3.40	7.28E-04
b2699	*recA*	Multifunctional DNA recombination and repair protein; ssDNA-dependent ATPase; synaptase; ssDNA and dsDNA binding protein forming filaments; ATP-dependent homologous DNA strand exchanger; recombinase A; LexA autocleavage cofactor	2.53	4.87E-05
b3175	*secG*	SecYEG inner membrane translocon secA-interacting subunit; preprotein translocase secAYEG subunit	2.43	3.43E-04
b4177	*purA*	Adenylosuccinate synthase, purine synthesis	2.37	6.70E-04
b4314	*fimA*	Fimbrin type 1, major structural subunit; phase variation	2.11	1.33E-03
b3231	*rplM*	50S ribosomal subunit protein L13; binds Zn(II)	2.00	1.12E-03
b3186	*rplU*	50S ribosomal subunit protein L21	1.92	5.10E-04
b3230	*rpsI*	30S ribosomal subunit protein S9	1.77	3.95E-04
b3739	*atpI*	ATP synthase, membrane-bound accessory factor	1.68	7.67E-04
b0406	*tgt*	tRNA-guanine transglycosylase; queuosine biosynthesis; zinc metalloprotein	1.66	1.97E-03
b3735	*atpH*	ATP synthase subunit delta, membrane-bound, F1 sector	1.66	1.70E-03
b4200	*rpsF*	30S ribosomal subunit protein S6; suppressor of dnaG-Ts	1.64	2.18E-03
b3409	*feoB*	Putative ferrous iron permease with GTP-binding domain	1.62	3.37E-03
b3810	*yigA*	DUF484 family protein, function unknown	1.57	2.66E-03
b0926	*ycbK*	Periplasmic M15A family non-protease, function unknown	1.56	8.53E-04
b0927	*ycbL*	Glyoxalase II homolog, function unknown	1.56	3.57E-03
b3704	*rnpA*	RNase P, C5 protein component; involved in tRNA and 4.5S RNA-processing	1.56	2.51E-03
b3342	*rpsL*	30S ribosomal subunit protein S12; RNA chaperone	1.54	2.60E-05
b4238	*nrdD*	Ribonucleoside-triphosphate reductase; class III anaerobic ribonucleotide reductase	1.54	1.83E-03
b1147	*ymfL*	Function unknown, e14 prophage	1.53	1.66E-03
b2150	*mglB*	D-galactose-, D-glucose-binding protein, periplasmic; substrate recognition for transport and chemotaxis; binds calcium	1.45	2.24E-03
b4148	*sugE*	Multidrug efflux pump; overexpression resistance to cetylpyridinium	1.44	1.22E-03
b3983	*rplK*	50S ribosomal subunit protein L11; kasugamycin sensitivity	1.44	2.33E-03
b0118	*acnB*	Aconitate hydratase 2; aconitase B; 2-methyl-cis-aconitate hydratase; iron-sulfur cluster; monomeric	1.42	1.80E-03
b0407	*yajC*	SecDFyajC membrane secretion complex subunit; assists the SecYE translocon to interact with SecA and export proteins	1.41	2.23E-03
b3357	*crp*	cAMP-activated global transcription factor; mediator of catabolite repression; CRP; CAP	1.40	1.49E-04
b2796	*sdaC*	L-serine:H+ symport permease, threonine-insensitive	1.40	1.84E-03
b3637	*rpmB*	50S ribosomal subunit protein L28	1.38	9.87E-05
b3508	*yhiD*	Predicted Mg(2+) transport ATPase, MgtC family, function unknown; inner membrane protein	1.35	2.62E-03
b3339	*tufA*	Translation elongation factor EF-Tu 1; GTP-dependent binding of aa-tRNA to the A-site of ribosomes; has intrinsic GTPase activity when bound to kirromycin	1.33	3.21E-03
b3985	*rplJ*	50S ribosomal subunit protein L10; streptomycin resistance	1.30	3.36E-03
b0428	*cyoE*	Cytochrome o oxidase protoheme IX farnesyltransferase subunit	1.26	8.29E-04
b0174	*ispU*	Undecaprenyl pyrophosphate synthase; dimeric	1.22	1.14E-04
b0756	*galM*	Aldose 1-epimerase, type-1 mutarotase; galactose mutarotase; monomeric	1.21	1.92E-03
b0946	*zapC*	FtsZ stabilizer	1.19	1.47E-04
b2279	*nuoK*	NADH:ubiquinone oxidoreductase subunit K, complex I; NADH dehydrogenase I	1.15	2.64E-04
b0388	*aroL*	Shikimate kinase II	1.12	6.85E-04
b4237	*nrdG*	Ribonucleotide reductase activase, generating glycyl radical; contains iron; binds NrdD tightly	1.11	4.09E-04
b4016	*aceK*	Isocitrate dehydrogenase kinase/phosphatase	1.11	1.10E-03
b1847	*yebF*	Extracellular Colicin M immunity family protein; function unknown	1.08	5.59E-04
b3961	*oxyR*	Oxidative and nitrosative stress transcriptional regulator	1.08	2.03E-03
b0463	*acrA*	AcrAB-TolC multidrug efflux pump; additionally dye, detergent, solvent resistance; membrane-fusion lipoprotein	1.06	2.36E-04
b3255	*accB*	Acetyl-CoA carboxylase, biotin carboxyl carrier protein; BCCP; homodimeric	1.05	6.41E-04
b0083	*ftsL*	Cell division and growth, membrane protein	1.01	9.70E-04
b1195	*ymgE*	UPF0410 family predicted inner membrane protein; function unknown	−1.01	2.15E-03
b0255	*insN'*	Pseudogene reconstruction, fused IS911 transposase AB	−1.08	2.42E-04
b4107	*yjdN*	Metalloprotein superfamily protein, function unknown	−1.30	4.90E-04
b1223	*narK*	Nitrate/nitrite antiporter; promotes nitrite extrusion and uptake	−1.31	3.47E-03
b1739	*osmE*	Osmotically inducible lipoprotein, function unknown	−1.46	1.01E-03
b0836	*bssR*	Repressor of biofilm formation by indole transport regulation; global regulator. e.g. of AI-2 transport and motility genes	−1.52	5.23E-05
b1795	*yeaQ*	UPF0410 family protein, function unknown	−1.56	1.65E-03
b2414	*cysK*	Cysteine synthase A, O-acetylserine sulfhydrylase A; homodimeric; selenate, azaserine, chromate resistance; alkali-inducible, sulfate starvation-inducible protein SSI5; cysteine desulfhydrase	−1.58	2.86E-03
b0806	*mcbA*	Stimulates colanic acid mucoidy, YhcN family, periplasmic; suppresses biofilm formation; repressed by McbR	−1.71	5.11E-04
b2425	*cysP*	Thiosulfate-binding protein, periplasmic	−1.84	1.90E-04
b0506	*allR*	Repressor for all (allantoin) and gcl (glyoxylate) operons; glyoxylate-inducible	−1.93	1.10E-03
b0456	*ybaA*	DUF1428 family protein	−1.98	2.30E-03

**Table 3:** RT-qPCR analysis of gene expression changes in 5-azacytidine treated and *dcm* knockout *Escherichia coli*

Gene	Phase	fc (log_2_) + aza/-aza (array)	fc (log_2_) + aza/-aza (RT-qPCR)	fc (log_2_) Δ*dcm*/wild-type (RT-qPCR)
*recA*	log	2.96	3.65^a^	0.68
*rsmI*	log	1.82	3.54^b^	2.86^a^
*atpH*	early stationary	1.66		0.64
*cspB*	early stationary	3.40		1.46^a^
*fimA*	early stationary	2.11		1.46^a^
*osmE*	early stationary	−1.46		−1.79^a^
*recA*	early stationary	2.53	2.98^b^	0.98
*rpoS*	early stationary	0.69	0.035	0.17
*osmE*	stationary			−3.0^a^
*rpoS*	stationary			−0.15

^a^fc (log_2_) > or <1 and *p* < 0.05 in qPCR experiment

^b^fc (log_2_) > or <1 and *p* < 0.005 in qPCR experiment

At logarithmic phase, the most obvious changes are genes that respond to DNA damage such as *recN*, *dinD*, *dinB*, and *dinG*. DAVID analysis indicates that the DNA repair and SOS response functional categories are overrepresented in this list (Table 4). We used a moderately stringent cut off for gene expression changes and there are other up-regulated SOS genes that have a p value <0.05, but did not make the FDR cutoff. One example is *recA*, which was shown to be up-regulated by RT-qPCR (Table 3). To determine if the gene expression changes are due to a direct effect of 5-azaC or loss of DNA methylation, we analyzed expression of *recA* and *rsmI* in a *dcm* knockout strain. *RecA* expression did not change in the *dcm* knockout strain indicating 5-azaC-dependent *recA* gene expression changes are not due to a loss of DNA methylation. However, expression of *rsmI*, a 16S rRNA methyltransferase, was increased in the *dcm* knockout strain, indicating *rsmI* expression is influenced by Dcm.

**Table 4:** Overrepresented functional categories in 5-azacytidine-treated *E. coli*

Stage	System	Name	p
logarithmic	GOTERM_BP_FAT	DNA repair	7.85E-05
logarithmic	GOTERM_BP_FAT	SOS response	8.10E-05
early stationary	KEGG Pathway	Ribosome	2.20E-10
early stationary	GOTERM_CC_FAT	Organelle inner membrane	7.40E-05

We further inspected the data for expression of DNA repair pathway genes in the presence of 5-azaC. The need for DNA repair after 5-azaC treatment is predicted to be due to the formation of DNA-Dcm crosslinks, as Dcm overexpression strains display increased sensitivity to 5-azaC induced killing [21, 28, 29, 32]. In addition, DNA repair may result in synthesis of new unmethylated DNA and explain methylation-dependent gene expression changes. At logarithmic phase, there is little evidence for 5-azaC induced expression of the mismatch repair genes *mutS*, *mutH*, and *mutL*, nucleotide excision repair pathway genes *uvrA*, *uvrB*, and *uvrC*, and pyrimidine base excision repair genes *ung, nth, mug,* and *mutM* (Table 1). We cannot rule out induction under conditions that were not evaluated. We did observe up-regulation of one gene that functions in translesion synthesis (*dinB*). The significance of increased *dinB* expression is unclear. Translesion synthesis is not thought to be required for 5-azaC induced DNA-protein crosslink repair as translesion mutants do not display increased sensitivity to the drug [21, 30, 32, 33]. Interestingly, three of the seven genes in Table 1 have a defined or predicted role in homologous recombination (*recN*, *rmuC*, *dinG*). Homologous recombination is thought to be the main pathway required for repair of 5-azaC induced damage as homologous recombination mutants are more sensitive to 5-azaC than wild-type strains [21, 28, 30, 32–34]. Thus, our data are consistent with a model where 5-azaC induced damage is repaired by homologous recombination, and components of this pathway are upregulated in 5-azaC treated cells.

At early stationary phase, there are 56 differentially expressed genes. Interestingly, the early stationary phase gene list is completely different from the logarithmic phase list (Tables 1 and 2). DAVID analysis identified functional categories ribosome and organelle inner membrane as overrepresented in the early stationary phase list (Table 4). Ribosomal protein genes have previously been reported as up-regulated in the *dcm* knockout strain [12, 13] and the organelle inner membrane list contains *sugE*, which has previously been reported to be up-regulated in the absence of *dcm* [11]. To identify new target genes impacted by cytosine DNA methylation, we analyzed a few targets in the *dcm* knockout strain via RT-qPCR (Table 3). We investigated *osmE* via RT-qPCR as it has a 5′CCWGG3′ site in its promoter (Fig. 3). *OsmE* expression decreases in the *dcm* knockout strain at early stationary phase, indicating Dcm influences *osmE* expression. *OsmE* was also down-regulated in the *dcm* knockout strain at stationary phase via RT-qPCR. Interestingly, *osmE* is the first example of a gene that is dependent upon Dcm for maximal expression. The expression of *atpH*, a subunit of ATP synthase, did not change in the *dcm* knockout strain, indicating the 5-azaC-dependent gene expression change is not due to a loss of DNA methylation. *FimA*, a fimbria subunit, was upregulated in the *dcm* knockout strain indicating control by Dcm. The expression of the *cspB* gene, a cold shock protein, increased in the *dcm* knockout strain, but not to the extent observed with 5-azaC treatment. Thus, *cspB* may respond to both the loss of DNA methylation and a 5-azaC-dependent DNA methylation-independent mechanism.

## Discussion

At logarithmic phase, the *dcm* gene is expressed as detected by microarray analysis and the DNA is methylated at 5′CCWGG3′ sites (Figs. 1 and 3). Yet there are very few 5-azaC-induced gene expression changes in the wild-type strain. The majority of 5-azaC changes are in DNA repair protein genes and are likely due to DNA damage and activation of the SOS response rather than a loss of DNA methylation (Table 4). 5-azaC-dependent activation of the SOS response has been reported [20, 21], and the pathway promotes survival in the presence of 5-azaC [20, 21, 28, 30, 32–34]. Although it is possible that we are missing *dcm*-dependent changes due to a moderately stringent statistical cutoff and/or the lack of 100 % methylation inhibition via 5-azaC, a simple model based on our data and three recent articles is that Dcm has only a minor effect on the logarithmic stage transcriptome [11–13]. It is noteworthy that the effect of Dcm on the transcriptome has only been tested under standard conditions (rich medium, 37 °C, aerobic, laboratory strain), and future work will evaluate other conditions.

At early stationary phase, the *dcm* gene is expressed as detected by microarray analysis, the DNA is methylated at 5′CCWGG3′ sites, and there are numerous 5-azaC-dependent gene expression changes. Not all of the genes induced by 5-azaC at stationary phase are predicted to be due to a loss of DNA methylation and future work will elucidate the mechanism of these gene expression changes not described in this article. A simple model based on our work and three recent studies is that Dcm has a significant impact on the early stationary phase transcriptome [11–13]. The mechanism of Dcm-dependent control of gene expression is not known and remains an enigma. Kahramanoglou et al. provided evidence that Dcm controls expression of the stationary phase specific RNA polymerase subunit *rpoS* gene and a loss of Dcm generates an increase in RpoS and RpoS-dependent gene products [13]. However, we did not observe up-regulation of *rpoS* expression in 5-azaC treated cells or *dcm* knockout cells via DNA microarrays and RT-qPCR (Tables 1, 2 and 3). What could explain the differences in *rpoS* expression in the absence of the *dcm* gene in the two studies? It is possible that genetic differences between the MG1655 strain used in the Kahramanoglou et al. study and the BW25113 strain used in this study could explain the differences in *rpoS* expression in the absence of *dcm*. The genome sequence of BW25113 was released in 2014 [35]. BLASTN searches using the MG1655 *rpoS* gene and 1000 basepair upstream region as a query indicated the same region is 100 % identical in BW25113 (data not shown). We also sequenced the main *rpoS* promoter responsible for stationary phase specific expression [36, 37], and there are no sequence differences between MG1655 and BW25113 (Additional file 3). We also sequenced four *rpoS* transcriptional start sites identified by Mendoza-Vargas *et al*. [38], and found no genetic differences between the two strains (Additional file 3). In summary, we have no evidence for genetic differences that potentially influence *rpoS* expression in MG1655 and BW25113. We certainly cannot rule out genetic changes outside of the *rpoS* loci in the two strains, epigenetic differences in the two strains, or posttranscriptional regulation of *rpoS* expression via Dcm.

Therefore, our data are not consistent with a model of Dcm-mediated regulation via RpoS and we must consider alternative models. What other molecules are stationary phase specific that could be effector molecules? One possibility is small RNAs, as some small RNAs are up-regulated in *E. coli* during stationary phase [39], and small RNAs are known to guide DNA methylation in plants [40]. Also, two of the best described Dcm-influenced genes, *sugE* and *osmE*, contain 5′CCWGG′3 sites near the -10 region of the promoter. Therefore, a model where Dcm directly influences gene expression by direct methylation of target genes still must be evaluated. DNA methylation could influence gene expression via numerous mechanisms including the alteration of DNA structure and influencing transcription factor binding. With respect to *osmE*, Regulon DB indicates the transcription factors FIS and IHF bind to the *osmE* promoter, but the binding sites do not overlap the 5′CCWGG3′ site [41]. It is important to note that ~11 % of *E. coli* promoters contain 5′CCWGG3′ sites [12]. Yet, most of these genes are not changing in response to DNA methylation loss, and therefore promoter methylation is not likely a common mechanism to control gene expression.

Since we observed gene expression changes in the absence of Dcm, another question concerns the biological consequences of the gene expression changes. As the vast majority of gene expression changes occur at early stationary phase, it is possible that Dcm has a role in stationary phase biology. During stationary phase of cells grown in liquid culture, ~99 % of the cells die within 7–10 days [42]. Subsequently, cells persist for long periods of time where waves of mutations can generate cells that can outcompete their predecessors (growth advantage at stationary phase, GASP). We are currently working to determine if Dcm promotes or reduces GASP.

## Conclusions

In this report, the *E. coli* transcriptomes of cells grown in the absence and presence of the cytosine DNA methylation inhibitor 5-azaC were compared. 5-azaC was found to be an effective DNA demethylating agent in *E. coli*. 5-azaC induced expression changes in 63 *E. coli* genes. The majority of the changes occurred at early stationary phase and were up-regulations. There are at least two mechanisms by which 5-azaC can induce gene expression changes. The first pathway is a DNA methylation-independent pathway that likely involves a DNA damage response. The second pathway is a DNA methylation-dependent pathway which occurs in the absence of changes in *rpoS* expression. The precise mechanism by which cytosine DNA methylation influences gene expression will be the focus of future studies.

## Methods

### Bacterial strains and growth

*E. coli* K-12 wild-type strain BW25113 and *dcm* knockout strain JW1944-2 have been previously described [12, 43]. The BW25113 (wild-type strain) genotype is F^−^, Δ(*araD*-*araB*)567, Δ*lacZ*4787(::rrnB-3), λ-, *rph*-1, Δ(*rhaD*-*rhaB*)568, *hsdR*415. The JW1994-2 (*dcm* knockout strain) genotype is F^−^, Δ(*araD*-*araB*)567, Δ*lacZ*4787(::rrnB-3), λ-, Δ*dcm-*735::*kan*, *rph*-1, Δ(*rhaD*-*rhaB*)568, *hsdR*415. Cells were grown in LB at 37 °C with shaking at 250 RPM to logarithmic phase (~2 h, A_600_ of ~0.45), early stationary phase (~8 h, A_600_ of ~2.7), and stationary phase (~24 h, A_600_ of 3.8). 5-azaC (Sigma) was dissolved fresh in 1x PBS at 5 mg/ml, sterilized by filtration through a 0.22 μM filter, and added to experimental samples at 0.5–50 μg/mL. The same volume of 1x PBS was added to control flasks.

### DNA methylation analysis via PspGI digestion

DNA was isolated from 2 mL of bacterial cells grown to logarithmic phase and early stationary phase using the Qiagen DNeasy kit. DNA quality and quantity was measured using a NanoDrop 1000 spectrophotometer. To assess methylation at 5′CCWGG3′ sites, DNA (0.5–1 μg) was incubated with 10 units of the restriction enzymes BstNI or PspGI for 2 h at 60 °C [12]. Reactions were monitored by electrophoresis on 1 % agarose gels and stained with 0.5 μg/mL ethidium bromide or 1x GelRed (VWR).

### Sodium bisulfite sequencing

1 μg of DNA was treated with sodium bisulfite according to the instructions in the EpiTect Kit (Qiagen). Bisulfite-treated DNA was used as a template for PCR. The conditions were 94 °C for 2 min, 94 °C for 1 min, 55 °C for 1 min, and 72 °C for 1 min for 30 cycles, followed by one cycle of 72 °C for 10 min. Primer sequences are osmE-F1-D 5′GAAAAGATAAAATTTTTTTAAAGTTAATT3′ and osmE-R1-D5′ACACTCAAAATTCCTACCATATTCTTATT3′. PCR products were inserted into the pGEM-T Easy plasmid (Promega), introduced into *E. coli* JM109, isolated using the alkaline lysis procedure (Wizard SV kit, Promega), and analyzed by Sanger sequencing (GeneWiz, New Jersey).

### Total RNA Isolation

At logarithmic phase, early stationary phase, and stationary phase, RNA was harvested from 4 mL of cells using the MasterPure RNA isolation kit according to the manufacturer’s instructions (EpiCentre). The optional DNase step was included in all preparations. RNA was analyzed by spectrophotometry on a NanoDrop 1000 spectrophotometer. For microarray experiments, RNA quality was assessed by bioanalysis on a RNA 6000 Nano chip at the University of Rochester Genomics Center. RIN values were between 8 and 10. For RT-qPCR experiments, RNA was analyzed by bioanalysis or traditional agarose gel electrophoresis after heating the RNA for 2 min at 65 °C in a solution containing 47.5 % formamide and 0.01 % sodium dodecyl sulfate.

### DNA Microarray analysis

Microarray experiments were performed with RNA from untreated cells and cells treated with 5 μg/mL 5-azaC. Total RNA was treated with the reagents in the mRNA Only Prokaryotic mRNA Isolation Kit to reduce rRNA levels and polyadenylate the mRNA according to the manufacturer’s instructions (EpiCentre). Modified RNA was used as input for array experiments. cRNA was made in the presence of either Cy3-CTP (RNA from untreated cells) or Cy5-CTP (RNA from 5-azaC-treated cells) using the Quick Amp 2-color Labeling Kit (Agilent) according to the manufacturer’s instructions. Equal amounts of dye-labeled cRNA were hybridized to *E. coli* 8*15 K DNA microarrays arrays (Agilent technologies). Microarrays were scanned on an Agilent Microarray Scanner. There were five biological replicates for both the logarithmic phase and early stationary phase samples. Data for non *E. coli* K-12 genomes on the array were removed prior to analysis. P values were determined with one-sample t-tests. The data were considered significant if the fold-change was > or < 2 fold and the Benjamini-Hochberg false discovery rate (FDR) was < 0.1. Pathway analysis was performed using DAVID with the high stringency setting and the same FDR cutoff [44].

### RT-qPCR analysis

RT-qPCR was performed as described previously [11]. Fold-changes were determined using the ΔΔCT method [45]. Three or more independent samples were used for each experiment for all targets except *fimA* (*n* = 2). The data were considered significant if the fold change was > or < 2-fold and the p value was <0.05 in one sample t-tests. Primer sequences are listed below.

- atpH-F, 5′TGCCGTCGAACACCAAAGTGTAGA3′
- atpH-R, 5′CAGTTGCTCACCACAAACTGCGAT3′
- cspB-F, 5′CGGCTTTATTTCTCCTGTTGATG3′
- cspB-R, 5′GGACCTTTAGCACCACTCTC3′
- fimA-F, 5′ACTCTGGCAATCGTTGTTCT3′
- fimA-R, 5′TGAACGGTCCCACCATTAAC3′
- mdh-F, 5′AAGTTGAAGTGCCGGTTATTGGCG3′
- mdh-R, 5′ATCAGCCACTTCCTGCTCGGTAAA3′
- osmE-F, 5′TGCGGGTAAACCTTCGTCTGAAGT3′
- osmE-R, 5′TTACTTCGCAGCCTGTGGATCAGT3′
- recA-F, 5′CGAAAGCGGAAATCGAAGGCGAAA3′
- recA-R, 5′TAGTGGTTTCCGGGTTACCGAACA3′
- rpoS-F, 5′CAGCCGTATGCTTCGTCTTA3′
- rpoS-R, 5′CGTCATCTTGCGTGGTATCT3′
- rsmI-F, 5′AGGGCCAGCTTTACATTGTACCGA3′
- rsmI-R, 5′TATCCTCGGCGGCAATCAGATCAA3′

### DNA sequencing analysis of the *rpoS* promoter

PCR was used to amplify the main stationary phase specific *rpoS* promoter [36, 37] and four *rpoS* transcription start sites [38]. *E. coli* BW25113 genomic DNA was used as a template and the Promega GoTaq master mix was used as a source of enzyme, dNTPs, and buffer. The conditions were 94 °C for 2 min, 94 °C for 1 min, 54 °C for 1 min, and 72 °C for 45 s for 30 cycles, followed by one cycle of 72 °C for 10 min. The PCR products were purified using the Qiagen QIAquick PCR purification kit and analyzed by Sanger DNA sequencing of both strands (GeneWiz, New Jersey). Three forward/reverse primer pairs were utilized; rpoSp-F1/rpoSp-R1, rpoSp-F2/rpoSp-R2, rpoSp-F3/rpoSp-R3. The primers sequences are listed below.

- rpoSp-F1, 5′ACCTTGCAGGTGGGTAATG3′
- rpoSp-R1, 5′GAGATAGGCGTACTGGTTGATG3′
- rpoSp-F2, 5′GCTACTCAACAACCGCAAATC3′
- rpoSp-R2, 5′CGTAGGCACTCAGGTAATCATC3′
- rpoSp-F3, 5′CAGCAAAGGACAGGCAATTATC3′
- rpoSp-R3, 5′CCTCAACTCCGTTCTCATCAA3′

### Statistical analysis

All statistical tests were performed in R [46].

## Abbreviations

5-azaC, 5-azacytidine; RT-qPCR, reverse transcription quantitative PCR

## Additional files

Additional file 1: Microarray fold-changes, p values, and false discovery rates (FDR) for all *E. coli* genes from logarithmic phase 5-azaC treated cells. Fold-change is expression in the presence of 5-azaC divided by expression in the absence of 5-azaC and is log_2_ transformed. P values are from one sample t-tests, and the FDR is the Benjamini-Hochberg false discovery rate. (CSV 153 kb) Additional file 2: Microarray fold-changes, p values, and false discovery rates (FDR) for all *E. coli* genes from early stationary phase 5-azaC treated cells. Fold-change is expression in the presence of 5-azaC divided by expression in the absence of 5-azaC and is log_2_ transformed. P values are from one sample t-tests, and the FDR is the Benjamini-Hochberg false discovery rate. (CSV 153 kb) Additional file 3: Sanger DNA sequencing analysis of the BW25113 *rpoS* promoter and transcription start sites. (PDF 509 kb)
