# Supplementary material for: 5-azacytidine induces transcriptome changes in Escherichia coli via DNA methylation-dependent and DNA methylation-independent mechanisms
Source: BMC Microbiol. 2016 Jun 27;16:130. doi: 10.1186/s12866-016-0741-4 (PMC4924334; doi:10.1186/s12866-016-0741-4)
Supplement: Additional file 3: — Sanger DNA sequencing analysis of the BW25113 rpoS promoter and transcription start sites. (PDF 509 kb) [file 12866_2016_741_MOESM3_ESM.pdf]

Additional file 3

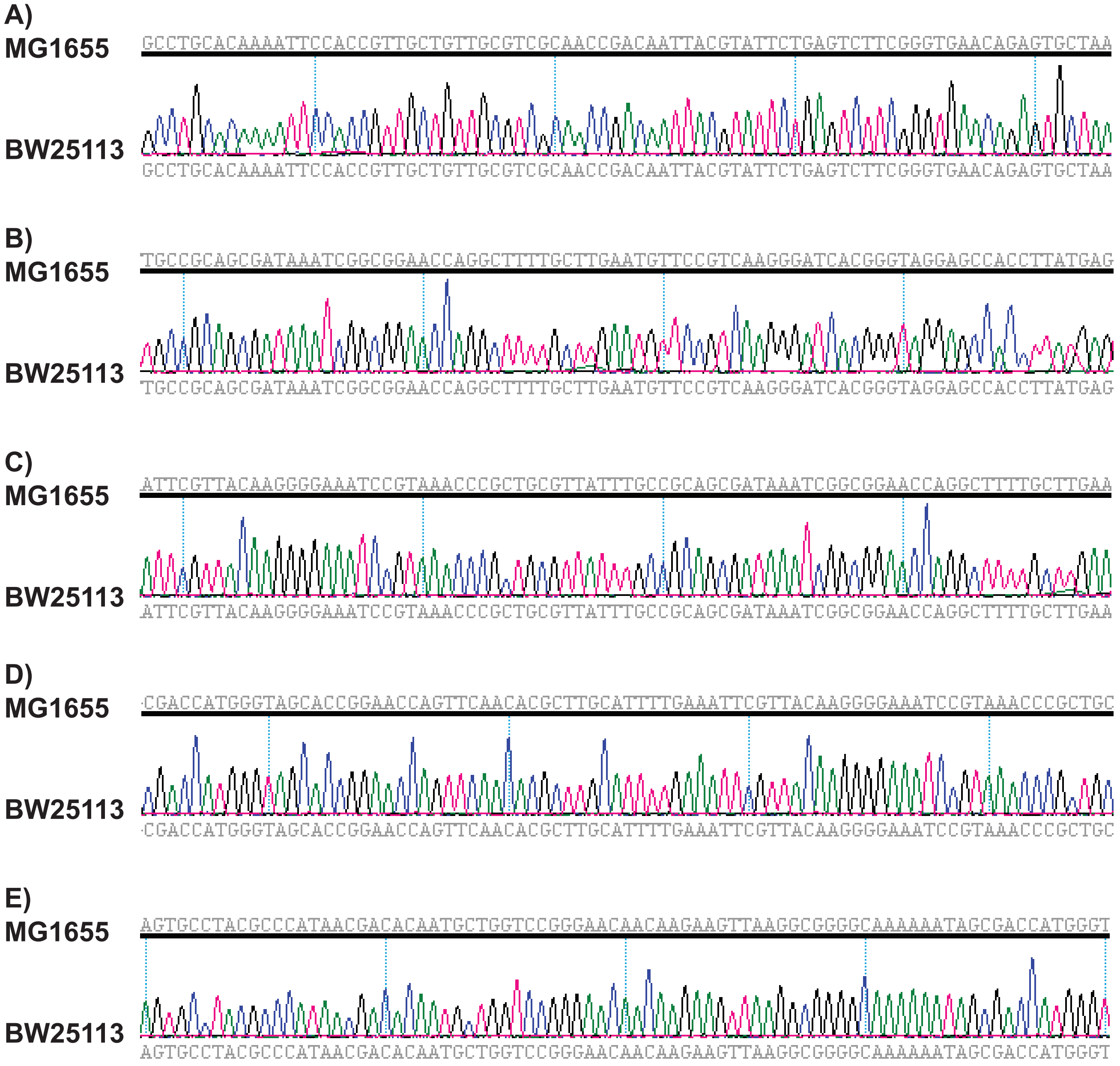

Additional file 3. DNA sequencing analysis of the *rpoS* promoter in *Escherichia coli* BW25113. The DNA specifying the main stationary-specific *rpoS* promoter and four *rpoS* transcription start sites was amplified by PCR and analyzed by Sanger DNA sequencing. In each diagram, the top sequence is the MG1655 sequence and the bottom sequence and corresponding chromatogram is the BW25113 sequence. A) represents the main *rpoS* promoter required for stationary phase transcription as described by Takayanagi *et al*. and Lange *et al*., and B-E) represent the four *rpoS* transcription starts sites (rpoSp1-rpoSp4) as described by Mendoza-Vargas *et al*.
